# Using SincNet for Learning Pathological Voice Disorders

**DOI:** 10.3390/s22176634

**Published:** 2022-09-02

**Authors:** Chao-Hsiang Hung, Syu-Siang Wang, Chi-Te Wang, Shih-Hau Fang

**Affiliations:** 1 Department of Electrical Engineering, Yuan Ze University, Taoyuan 320, Taiwan; 2 Department of Otolaryngology Head and Neck Surgery, Far Eastern Memorial Hospital, New Taipei City 220, Taiwan

**Keywords:** pathological voice, classification, sinc functions, convolutional neural network, SincNet

## Abstract

Deep learning techniques such as convolutional neural networks (CNN) have been successfully applied to identify pathological voices. However, the major disadvantage of using these advanced models is the lack of interpretability in explaining the predicted outcomes. This drawback further introduces a bottleneck for promoting the classification or detection of voice-disorder systems, especially in this pandemic period. In this paper, we proposed using a series of learnable sinc functions to replace the very first layer of a commonly used CNN to develop an explainable SincNet system for classifying or detecting pathological voices. The applied sinc filters, a front-end signal processor in SincNet, are critical for constructing the meaningful layer and are directly used to extract the acoustic features for following networks to generate high-level voice information. We conducted our tests on three different Far Eastern Memorial Hospital voice datasets. From our evaluations, the proposed approach achieves the highest 7%–accuracy and 9%–sensitivity improvements from conventional methods and thus demonstrates superior performance in predicting input pathological waveforms of the SincNet system. More importantly, we intended to give possible explanations between the system output and the first-layer extracted speech features based on our evaluated results.

## 1. Introduction

In recent decades, automatic detection of voice pathologies gathered a lot of academic interest because such voice disorders are one of the most popular health issues [1]. The literature in [2] has reported that nearly 30% of the general population encountered voice disorder problems. Due to the popularity of smartphones and smart speakers, the most convenient way for screening voice disorders may be using such end devices which contain the acoustic signals storage functions [3]. With automatic detection technology, voice disorders can be analyzed using the collected acoustic signals either in clouds or end devices. The main advantage of this approach is that it can greatly reduce unnecessary medical demands because patients can be filtered who truly need hospital services at the beginning stage. Furthermore, this can effectively minimize the contact possibility. That would be an alternative advantage of this approach, especially during the COVID-19 pandemic, and thus motivates many researchers to investigate speech signals for pathologies detection and classification tasks in recent years [4,5,6].

The voice pathologies detection problem is of paramount importance to many healthcare applications. To address this issue, the essential acoustic input signals are usually decomposed into several voice representations [7], such as cepstrum [8,9,10], jitters [11], and entropy [12]. Meanwhile, these features are then handled by a pathological detection system, which is constructed in terms of machine-learning techniques, including a k-nearest neighbor, and hidden Markov model classifiers [13]. Even though these techniques can provide decent recognition accuracy, the performance can be further promoted by applying novel deep-learning approaches. For example, a series of deep learning neural networks and their variations were investigated for such pathological voice detection tasks [14,15,16,17,18,19]. In [20], domain adaptation methods were also applied to deal with the device variation issues [20]. In addition to the research methods, the voice database gathered a lot of academic interest. For instance, IEEE Big Data held an international and public competition, namely FEMH-Challenge. In this challenge, Far Eastern Memorial Hospital (FEMH), Taiwan [21,22] has released a dataset, containing a hundred acoustic waveforms from different voice disorders. Unlike the other pubic pathological voice database, MEEI [23] and SVD [24], this dataset is relatively small but with more detailed attributes. In addition, this challenge builds a fair and systematic evaluation protocol, and over one hundred research groups worldwide are involved in this international voice disorders detection competition [9,10,11,25,26,27,28].

Since the rapid development of artificial intelligence, deep learning has played an important role in many data-driven applications [3,5,6,16,17,18,19]. Although deep learning can learn complex and abstract feature representations from data, the main disadvantage is that the trained model lacks interpretability [29,30,31]. Until recently, deep learning algorithms have been notorious for being black boxes, making it difficult to explain results insights and to understand their inner processes. This lack of explainability may be a vital bottleneck hindering the development of deep learning technology [32,33]. In addition, model interpretability is extremely important in specific domains, such as healthcare applications. Without sufficient model interpretability, the hearth care systems might not be legally permitted to use [34]. The flaws of applying deep-learning techniques to detect pathological voices motivate us to explore the possibility of explainable models in this study and to provide interpretability while learning the pathological voice.

As mentioned above, for processing sound timing signals, the extraction ability of the first layer in neural networks is important because this is the first step that directly processes speech from the raw waveform [35]. That represents the effectiveness of the low-dimensional features extracted by the first layer and the premise for the high-level network to learn meaningful high-dimensional feature information. In this study, SincNet [35,36] is used to learn the pathological voice to provide more model interpretability. Unlike the traditional convolutional neural network (CNN) model, SincNet exploits parametrized sinc functions to replace the first layer in CNN, encouraging the first layer to discover more meaningful filters. In this way, only low and high cutoff frequencies of band-pass filters are directly learned from data, and it offers a compact way to derive a filter-bank bandwidth with a clear physical meaning. Notably, the SincNet approach has been investigated in speaker identification and verification tasks [35]. To our knowledge, this is the first work to use SincNet for learning pathological voice disorders. Our experiments, conducted on the FEMH voice disorders dataset, show that the proposed architecture converges faster, performs better, and is more interpretable than standard CNN. Results show that SincNet provided improved performance in different experimental setups. The learned power spectral density reveals that SincNet learns more details than CNN.

## 2. Materials and Methods

### 2.1. Database Description

Our evaluations were conducted on the FEMH Speech Disorders database, where all speech signal recordings were collected by Far Eastern Memorial Hospital’s Speech Clinic from 2012 to 2019. There was 1,061 samples in /a/-voices. Each /a/ sound is about three seconds long. This work focused on explainable ability improvement compared to existing works. That is why we use the vowel ‘/a/’ sound, which is a typical speech in literature because this sound is language-independent. In Table 1 the distribution columns of sound samples for neoplasm (Neo), functional dysphonia (FD), vocal palsy (VP), and phonotrauma (Pho) is shown. All waveforms were recorded using high-quality microphones and digital amplifiers at background noise levels of 40 to 45 dBA and were recorded at a sampling rate of 44,100 Hz and 16-bit integer resolution. For each /a/ sound corpus, the data were split using an 8:2 approach, with 80% of the sounds used to form the training and validation sets (i.e., 848 sounds selected from the /a/ sound database, respectively), and the remaining 20% of the sounds used to provide the test set. It is worth noting that there is no overlap between the training and test sets.

**Figure 1:**
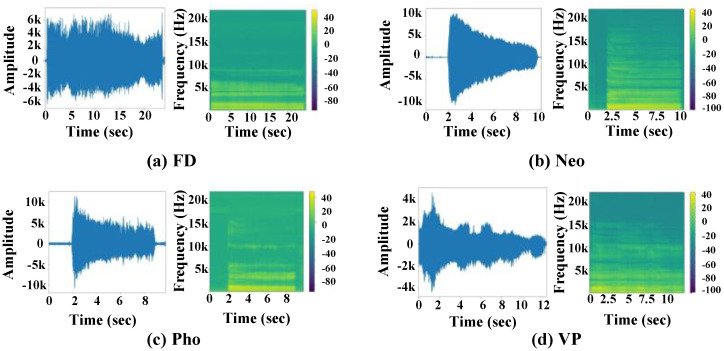
Waveform and Spectrogram of pathological voices.

Figure 1 was depicted to visualize pathological utterances in terms of the vowel /a/ sound. In this figure, we illustrated FD, Neo, Pho, and VP voices in Figure 1a, b, c, and d, respectively. We placed the waveform in the left panel for each subfigure, while the associated spectra were put on the right side. In addition, we listed the color bar next to the spectra to illustrate the energy of the magnitude in the frequency domain. From Figure 1a, the FD voice, which is dysphonia, but normal on endoscopy, provides the stationary amplitude of the waveform envelop along the time axis. A tumor in the larynx with hoarseness, the main symptom, introduces many high-frequency components of a Neo voice in Figure 1b. The sound structures of Pho in Figure 1c, which is the combination of nodules, polyps, and cysts, show an intermediate interference in high-frequency parts. Meanwhile, in Figure 1d, the sound of those VP patients, who cannot maintain the opening and closing of their vocal cords, results in the nonstationary energy trajectory while pronouncing the /a/-vowel.

Another dataset published by an international competition (called the FEMH challenge) is adopted in the experiments for further evaluation. The statistical data collation of the FEMH-Challenge database can be seen in Table 2 and Table 3. From Table 2, we can find that this database is relatively smaller than Table 1, but more balanced among the three categories. We were able to make a comparison with the existing methods from different perspectives. More specifically, this database consists of 150 /a/ vowels, which are pronounced by 150 different patients and are divided into Neo, Pho, and VP, as well as 50 normal sounds. The front-end data collection procedures, environment, and separation of the datasets are identical.

From Table 3, we can find that this database is relatively smaller than Table 1, but more balanced among the four categories. We were able to make a comparison with the existing methods from different perspectives.

### 2.2. The SincNet Architecture

**Figure 2:**
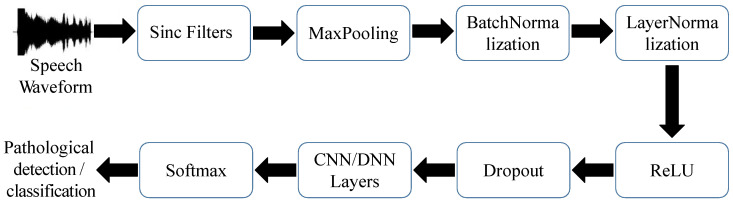
Architecture of SincNet.

In this study, the block diagram of the applied SincNet is listed in Figure 2, which comprises a series of parametrized sinc filters, normalization layers, and the conventional CNN module. For the first single processing layer, an input waveform is first normalized and processed by a temporal sinc function, a band-pass filter formulated in Equation (1) concerning parameters $f_{1}$ and $f_{2}$. In addition, the $f_{1}$ and $f_{2}$ represent the filter’s lowest- and highest-cutoff frequencies, respectively, and are learnable factors for discovering more meaningful pathological structures during the training process. Consequently, any band information of an input single within these frequencies is preserved for the following layers without distortions.

(1) $$g\left[n,f_{1},f_{2}\right]=2f_{2}sinc\left(2\pi f_{2}n\right)-2f_{1}sinc\left(2\pi f_{1}n\right).$$

After passing a signal through sinc filters, these acoustic features are collected and handled by a standard CNN pipeline (max-pooling, normalization, activations, dropout, CNN). Finally, we leverage the softmax function for identifying/classifying pathological voices. There are 80 sinc functions (160 learnable parameters) with each filter a length of 251, and then it has two stacked conventional convolutional layers with each layer the parameter size [60, 5], which follow the setting representation “[channel size, kernel size]”. Thereafter, we applied three 2048-node fully connected (FC) layers. Finally, a Softmax layer provides the set of posterior probabilities for the classification or detection. Notably, we applied 80 sinc functions to extract voice features in this task to align with the conventional studies that 80-dimensional acoustic features are sufficient to represent the articulatory characters of an input voice [37,38]. In addition, our preliminary tests also show that applying 80 sinc functions to the input layer provides the best system performance. Between hidden layers, we performed nominations to shrink the possible gradient vanishing issue and applied leaky Relu activation to regularize the associated output. The dropout rate for CNN was set to 0.5, while that for DNN was 0.3.

### 2.3. The DNN Architecture

In addition to SincNet, we implemented two different CNN models as the baseline systems for this task. The first model structure of CNN (denoted as “CNN(1D)” in this study) is the same as those of SincNet. More preciously, the [channel size, kernel size] in the first convolutional layer is [80, 251], and then followed by two [60, 5] ([channel size, kernel size]) convolutional and three 2048-node FC layers. The result propagation, activation function, and Softmax layer structures were exactly the same between SincNet and CNN in the following experiments. Meanwhile, the second CNN model (represented as “CNN(2D)” for the following sections) comprises six hidden layers. The first three convolutional layers are performed to process the input waveform, in which the [channel size, kernel size, strides] is [80, (3, 3), (3, 3)], [60, (3, 3), (3, 3)], and [60, (3, 3), (3, 3)] in order. For each convolution layer, the maximum pooling with pool size being (3, 3), the batch and layer normalizations are then applied right after performing the convolution process to the layer input. In addition, the dropout operator was then used in the latest two convolutional layers (the dropout rates were 0.5 and 0.4, respectively) to improve the system robustness. After that, the flattened and three FC layers were then carried out to handle the outcome of the convolution model. In each FC layer, there are 2048 nodes, followed by the Batch, Layer normalizations, and dropout process. The dropout rates were individually set for these FC layers to 0.3, 0.2, and 0.2. Notably, the difference between conventional CNN and SincNet is in the first layer, in which SincNet uses two parameters ($f_{1}$ and $f_{2}$) for representing a sinc filter, while CNN learns all kernel parameters to extract voice features.

In this pathological detection/classification task, we use the 5-fold cross-validation method for evaluating SincNet and CNN. Details of the CNN model training and parameters optimization can be found in previous publications [39].

### 2.4. Evaluation Metrics

We formulated the accuracy, sensitivity, and unweighted average recall (UAR) metrics in Equation (2). These metrics were leveraged to demonstrate the performance of used SincNet and CNN models and were derived in terms of the combination of true positive (TP), true negative (TN), false positive (FP), and false negative (FN) values. Among these evaluation metrics, the accuracy was used to evaluate the correctness of the true prediction overall testing samples (TN+TP+FN+FP), while the sensitivity performs the recall rate of a specific class D∈{FD,Neo,Pho,VP}, which was calculated by dividing the correct predictions from the size of all positive-condition samples (${\mathrm{TP}}_{D}+{\mathrm{FN}}_{D}$). The UAR is the average sensitivity on all numbers of classes (#D).

(2) $$\begin{array}{cc} \mathrm{Accuracy} & =100\%\times \frac{\mathrm{TN}+\mathrm{TP}}{\mathrm{All}\mathrm{testing}\mathrm{samples}},\\ {\mathrm{Sensitivity}}_{D} & =100\%\times \frac{{\mathrm{TP}}_{D}}{{\mathrm{TP}}_{D}+{\mathrm{FN}}_{D}},\\ \mathrm{UAR} & =100\%\times \frac{\sum_{D}{\mathrm{Sensitivity}}_{D}}{\#D}, \end{array}$$

where, #D=4 was used for FEMH, while #D=3 was set for FEMH-Challenge datasets in this study.

In addition to typical evaluation metrics, this study also uses t-SNE to visualize the training results. t-SNE is a non-linear machine learning dimensionality reduction method proposed by Laurens van der Maaten and Geoffrey Hinton in 2008 [40]. The ability of t-SNE to preserve local structure during dimensionality reduction. It has become a regular tool for data visualization and modeling competitions in recent years. The advantages and details of the t-SNE computation can be found in [41].

## 3. Experimental Results

In this section, three deep learning classifiers (CNN(1D), CNN(2D), and SincNet) were implemented using FEMH /a/-vowel training corpus to detect and classify vocal disorders. The FEMH data includes two public data sets, the 2018 and 2019 FEMH Challenge, wherein different voice disorders tasks were compared to show the ability of SincNet. The details of the experimental results were described in the following subsections.

### 3.1. Voice Disorders Detection

This section first investigates voice disorders detection using the 2018 FEMH Challenge Detection dataset. Table 4 shows four performance metrics, sensitivity, specificity, accuracy, and UAR under /a/-vowel testing conditions. The results show that CNN(1D) slightly outperforms CNN(2D). More importantly, SincNet performs the best, achieving the highest sensitivity of 80.00% while keeping the highest specificity of 65.00%. Both standard deviations are the lowest in these indicators. The results also show that SincNet provided 77.50% in accuracy and 72.50% in UAR, demonstrating the best performance compared to other CNN-based detection systems. This result indicates that SincNet successfully extracts robust features from /a/-vowels even in the face of a small data challenge, thus improving the effectiveness of the voice disorder detection task.

**Figure 3:**
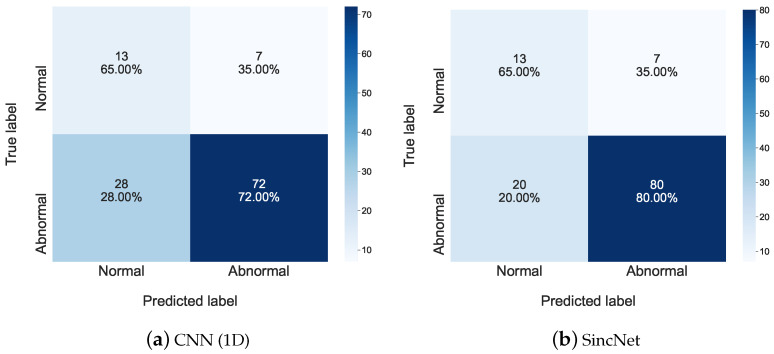
The confusion matrix is used to show detailed detection performance of (**a**) CNN(1D) and (**b**) SincNet on the 2018 FEMH Challenge dataset.

Confusion matrices of the same detection task using the 2018 FEMH Challenge dataset were illustrated in Figure 3. We listed the detection results of CNN(1D) in Figure 3a, and those of SincNet in Figure 3b. In addition, the horizon and vertical axis of each sub-figure represented the predicted and true labels, respectively. For each figure, we showed the number of predictions and their percentage under the associated true condition in a block. From the figure, the sensitivity to the abnormal condition of CNN(1D) was 72.00%, while that of SincNet was 80.00%. The higher sensitivity score of SincNet indicates that the applied Sinc Filters can effectively extract voice features from abnormal speeches, thus improving the detection accuracy of CNN(1D) for this detection task.

**Figure 4:**
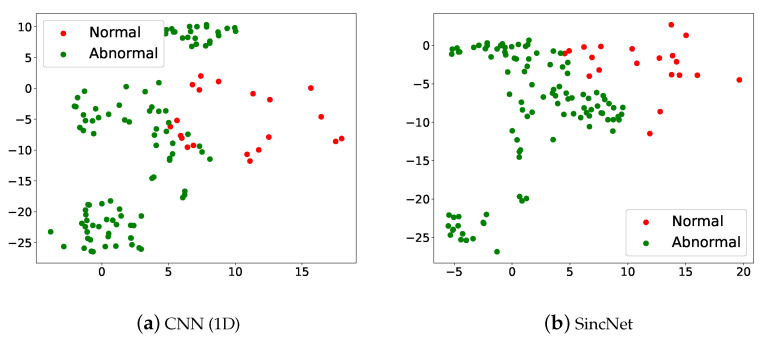
Scatter plots of (**a**) CNN(1D) and (**b**) SincNet show the t-SNE performance for detecting normal and abnormal voice on the 2018 FEMH Challenge dataset.

We perform t-SNE to further demonstrate the detection performance of CNN1D and SincNet in Figure 4. The figure was made in the following steps: (1) We placed an input /a/-vowel voice at the input side of a model and passed it across the entire detection system. (2) The sentence-level feature was then derived from the input of Softmax. (3) Finally, we collected all utterance-level acoustic features and performed a t-SNE analysis. The result of classifying the /a/-vowel through machine learning is converted into two-dimensional grouping by t-SNE. Each point in the graph represents a person with/without a voice disorder. Red dots are normal, green dots are abnormal. Looking at Figure 4, it can be seen that the two types of voice disorders can be clearly distinguished, but there is some overlap.

Table 5 illustrates the averaged accuracy scores of detection, respectively under /a/-vowel testing conditions. The results confirm that CNN(1D) outperforms CNN(2D), and SincNet in turn outperforms CNN(1D). SincNet successfully extracts robust features from /a/-vowels, thus improving the effectiveness of the speech impairment Detection task. The overall performances of various baselines and the proposed method. The sensitivities for each disorder, including normal and abnormal, were presented and analyzed. We verify the effectiveness of SincNet layers. Results show that the SincNet outperforms CNN. The results show that SincNet provided 83.33% in accuracy and 77.31% in UAR, demonstrating the best performance compared with other Detection systems.

**Figure 5:**
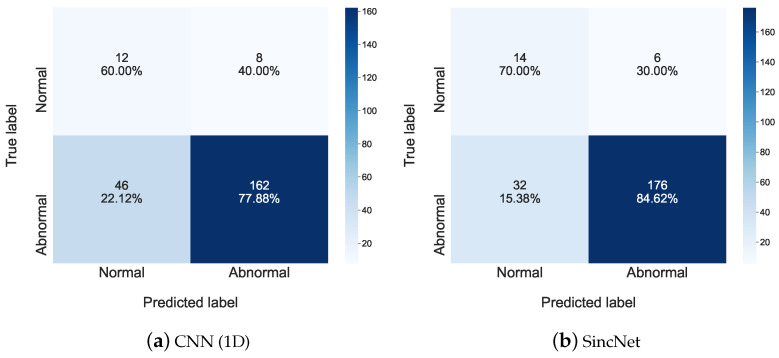
The confusion matrix is used to show detailed detection performance of (**a**) CNN(1D) and (**b**) SincNet on the FEMH dataset.

We presented the confusion matrix of the FEMH dataset, for all the traits of the detection task in Figure 5. Similar to Figure 3, the percentage in each block denoted the ratio between the prediction numbers and the whole samples in a true condition. From the figure, it is possible to observe that most errors committed by the system occur in the labels adjacent to the diagonal, affecting labels normal and abnormal. We can also observe that the sensitivity to the abnormal condition of SincNet (84.62%) was higher than that of CNN(1D) (77.88%). In addition, the sensitivity result to the normal condition in Figure 5a was 60.00% and that value in Figure 5b was 70.00%. These results confirm again the effectiveness of applying Sinc Filters to extract acoustic features and help a detection system distinguish pathological voices from normal ones.

**Figure 6:**
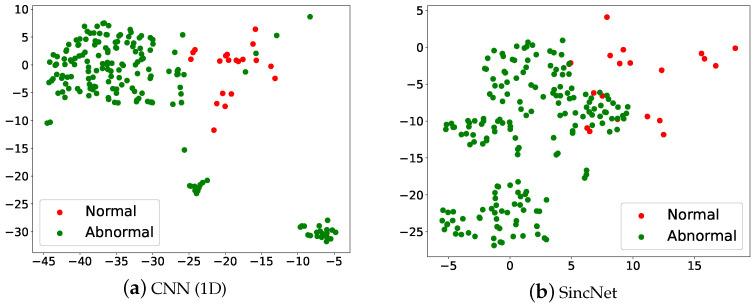
Scatter plots of (**a**) CNN(1D) and (**b**) SincNet show the t-SNE performance for detecting normal and abnormal voice on the FEMH dataset.

Figure 6 illustrates the t-SNE Clustering, Red dots are normal, and green dots are abnormal. We can see that the two kinds of voice disorders can be clearly distinguished. But there is a partial overlap between normal and abnormal, which may also be verified from Figure 5. Results show that the SincNet outperforms the CNN.

### 3.2. Classification 3 Class

Table 6 illustrates the averaged accuracy scores of classification under /a/-vowel testing conditions. The results confirm that CNN(1D) outperforms CNN(2D), and SincNet in turn outperforms CNN(1D). In the classification task that divided the samples into three categories, SincNet achieved the highest accuracy (73.00%) and UAR (74.03%). The results confirm that the SincNet successfully extracts the robust features from /a/-vowel, thus improving the effectiveness of the voice disorders classification task.

**Figure 7:**
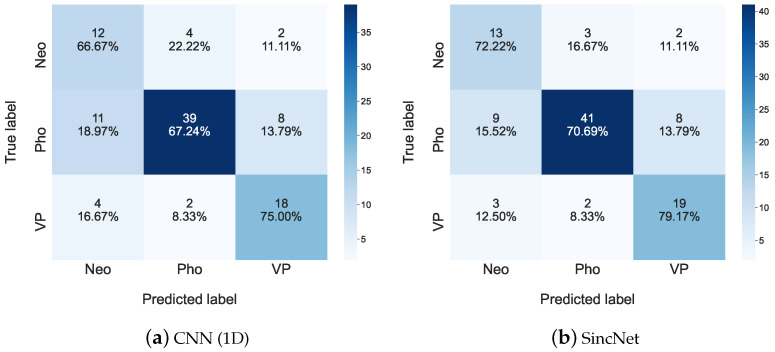
Confusion matrices for (**a**) CNN(1D) and (**b**) SincNet are used to show detailed classification performance among Neo, Pho, and VP on the 2018 FEMH Challenge dataset.

Figure 7 depicts the detailed classification results of the 2018 FEMH Challenge, for all the traits of the classification task. For each sub-figure in Figure 7, the performance in the diagonal is higher than those in other metric elements. This observation suggests that both CNN(1D) and SincNet can be applied to identify the voice-disorder type. Let us take a closer look at the classification results between Neo and Pho, there is 18.97% from predicting true Pho as Neo, and 22.22% from misclassifying true Neo to Pho in Figure 7a; those errors were then reduced to 15.52% and 16.67%, respectively from the SincNet classification system in Figure 7b. These results demonstrate that SincNet is able to reduce the error prediction between the Neo and Pho voice disorders.

**Figure 8:**
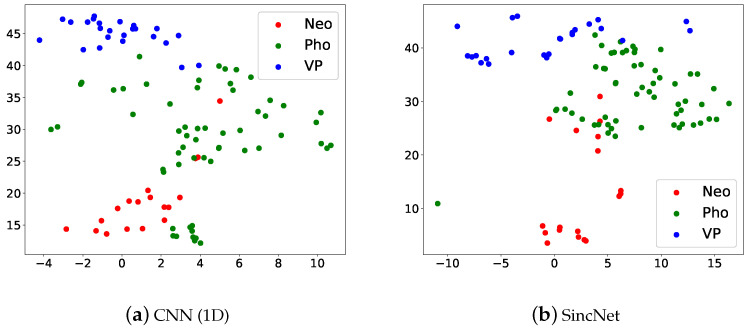
Scatter plots of (**a**) CNN(1D) and (**b**) SincNet show the t-SNE performance for classifying Neo, Pho, and VP on the 2018 FEMH Challenge dataset.

Figure 8 illustrates the t-SNE Clustering, Red dots are Neo, green dots are Pho and blue dots are VP. We can see that the three kinds of voice disorders can be distinguished. But there is a partial overlap between Pho and the other two categories, which may also be verified from Figure 7. Results show that the SincNet outperforms CNN.

We remove FD sounds in the 2019 FEMH Challenge to provide a fair comparison with different perspectives. Table 7 illustrate the averaged accuracy scores of classification, under /a/-vowel testing conditions. The results confirm that CNN(1D) outperforms CNN(2D), and SincNet in turn outperforms CNN(1D). In the classification task that divided the samples into three categories, SincNet achieved the highest accuracy (70%) and UAR (70%). The results confirm that the SincNet successfully extracts the robust features from /a/-vowel, thus improving the effectiveness of the voice disorders classification task.

**Figure 9:**
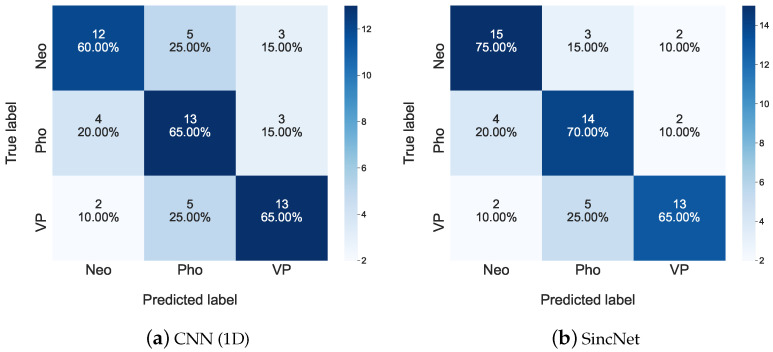
Confusion matrices for (**a**) CNN(1D) and (**b**) SincNet are used to show detailed classification performance among Neo, Pho, and VP on the 2019 FEMH Challenge dataset.

We then list the detailed predicted performance among Neo, Pho, and VP for the 2019 FEMH Challenge in Figure 9. Each diagonal element in Figure 9a or b shows the highest ratio along the corresponding column. In addition, we can observe that the CNN(1D) system provides 25.00% errors from misclassifying those samples with true Neo label to be Pho one. This error rate is largely decreased to 15.00% in Figure 9b. These evaluations imply the decent feature-extraction capability of the used Sinc Filters and the effectiveness of the SincNet classification system in reducing the predicting error between Neo and Pho.

**Figure 10:**
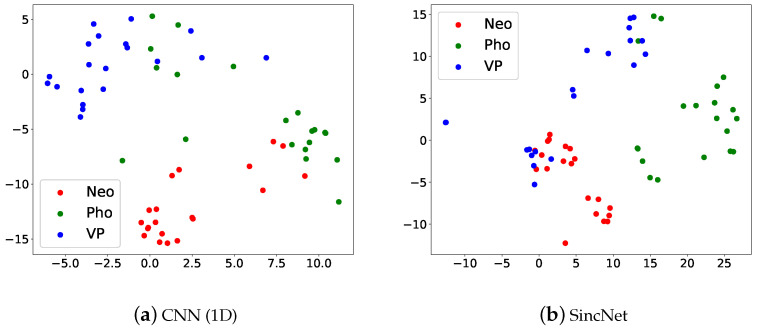
Scatter plots of (**a**) CNN(1D) and (**b**) SincNet show the t-SNE performance for classifying Neo, Pho, and VP on the 2019 FEMH Challenge dataset.

In the 2019 FEMH Challenge, we remove the FD sound. Figure 10 illustrates the t-SNE Clustering, Red dots are Neo, green dots are Pho and blue dots are VP. We can see that the three kinds of voice disorders can be distinguished. But there is a partial overlap, which may also be verified from Figure 9. Results show that the SincNet outperforms CNN.

In FEMH we remove FD sound. Table 8 illustrate the averaged accuracy scores of classification, under /a/-vowel testing conditions. The results confirm that CNN(1D) outperforms CNN(2D), and SincNet in turn outperforms CNN(1D). In the classification task that divided the samples into three categories, SincNet achieved the highest accuracy (81.28%) and UAR (80.05%). The results confirm that the SincNet successfully extracts the robust features from /a/-vowel, thus improving the effectiveness of the voice disorders classification task.

**Figure 11:**
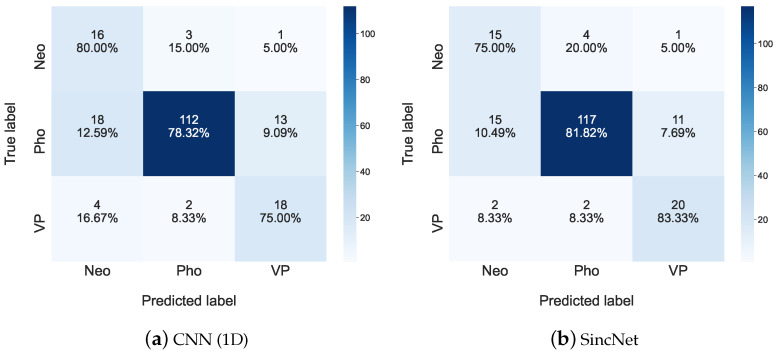
Confusion matrices for (**a**) CNN(1D) and (**b**) SincNet are used to show detailed classification performance among Neo, Pho, and VP on the FEMH dataset.

Figure 11 illustrates the predicted results among Neo, Pho, and VP for the FEMH dataset. The diagonal components of each sub-figure in Figure 11 show the best performance over other elements. However, when comparing the classified performance in the top row of both Figure 11a,b, the sensitivity for Neo is degraded from 80.00% to 75.00%, while the error classification ratio between true Neo and predicted Pho is increased from 15.00% to 20.00%. One possible inference for this phenomenon is the predicted variance of a model. Meanwhile, the sensitivity of the VP is 75.00%, which is generated by CNN(1D) and is improved to 83.33% which is provided by SincNet. The above observations not only confirm again the decent classification capability of SincNet but imply the acoustic properties of VP are much different from those of Neo, or Pho.

**Figure 12:**
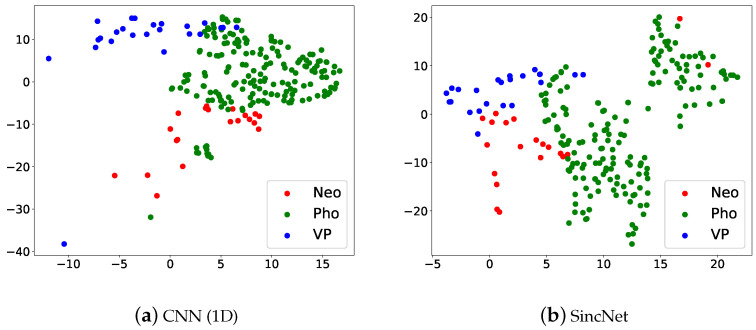
Scatter plots of (**a**) CNN(1D) and (**b**) SincNet show the t-SNE performance for classifying Neo, Pho, and VP on the FEMH dataset.

In FEMH we remove FD sound. Figure 12 illustrates the t-SNE Clustering, Red dots are Neo, green dots are Pho, and blue dots are VP. We can see that the three kinds of voice disorders can be distinguished. But there is a partial overlap between Pho and the other two categories, which can be verified from Figure 11. Results show that the SincNet outperforms CNN.

### 3.3. Classification 4 Class

Table 9 illustrates the averaged accuracy scores of classification under /a/-vowel testing conditions. The results confirm that CNN(1D) outperforms CNN(2D), and SincNet in turn outperforms CNN(1D). In the classification task that divided the samples into three categories, SincNet achieved the highest accuracy (68.75%) and UAR (68.75%). The results confirm that the SincNet successfully extracts the robust features from /a/-vowel, thus improving the effectiveness of the voice disorders classification task.

**Figure 13:**
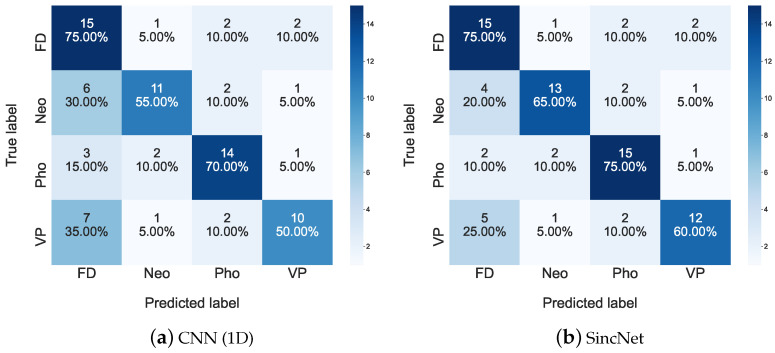
Confusion matrices for (**a**) CNN(1D) and (**b**) SincNet are used to show detailed classification performance among FD, Neo, Pho, and VP on the 2019 FEMH Challenge dataset.

The confusion matrix of the 2019 FEMH Challenge is presented in Figure 13. In this figure, we list the results between predictions and true FD, Neo, Pho, and VP labels. In addition, the performance in Figure 13a was provided by the CNN(1D) classification system, while those in Figure 13b was depicted based on SincNet predicted outcomes. In the first column of Figure 13a, those results, which were predicted to be FD by CNN(1D), comprise 16 misclassified samples (7 belong to VP, 3 for Pho, and 6 are Neo). Those misclassifications are then reduced to 11 samples in Figure 13b. The observation indicates that the applied Sinc Filters for input /a/-sounds can distinguish FD from VP, Pho, and Neo voice-disorder functions and thus improve the Accuracy and UAR of SincNet from CNN(1D) in Table 9.

**Figure 14:**
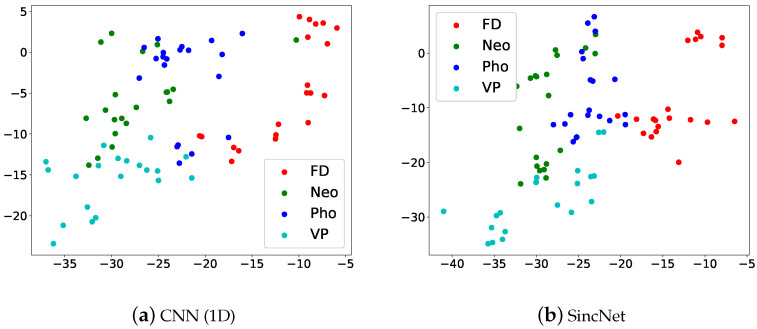
Scatter plots of (**a**) CNN(1D) and (**b**) SincNet show the t-SNE performance for classifying FD, Neo, Pho, and VP using 2019 FEMH Challenge dataset.

Figure 14 illustrates the t-SNE Clustering, Red dots are FD, green dots are Neo, blue dots are Pho and light blue dots are VP. We can see that the four kinds of voice disorders can be clearly distinguished. But there is a partial overlap, which may also be verified from Figure 13. Results show that the SincNet outperforms CNN.

Table 10 illustrates the averaged accuracy scores of classification under /a/-vowel testing conditions. The results confirm that CNN(1D) outperforms CNN(2D), and SincNet in turn outperforms CNN(1D). In the classification task that divided the samples into three categories, SincNet achieved the highest accuracy (71.01%) and UAR (64.30%). The results confirm that the SincNet successfully extracts the robust features from /a/-vowel, thus improving the effectiveness of the voice disorders classification task.

**Figure 15:**
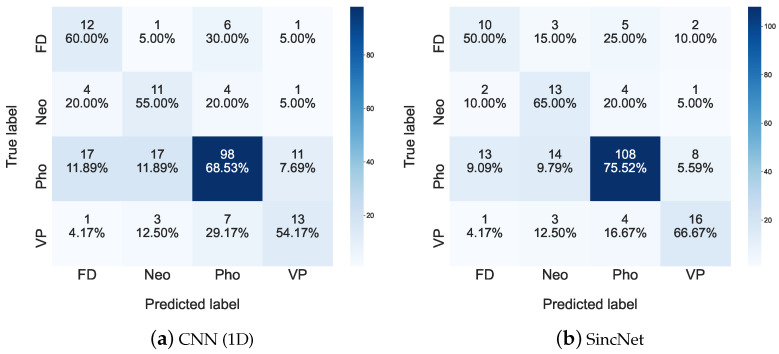
Confusion matrices for (**a**) CNN(1D) and (**b**) SincNet are used to show detailed classification performance among FD, Neo, Pho, and VP on the FEMH dataset.

Figure 15 depicts the confusion matrix of the FEMH for all the traits of the classification task. From the figure, the sensitivities of Neo, Pho, and VP that are performed by testing SincNet in Figure 15b are increased when comparing those of CNN(1D) in Figure 15a. In addition, given a specific true condition, the number of misclassified samples that CNN introduced (1D) in Figure 15a are decreased when compared with those provided by the SincNet system in Figure 15b except the evaluation for the true FD condition. These results confirm again the superior performance of the SincNet classification system.

**Figure 16:**
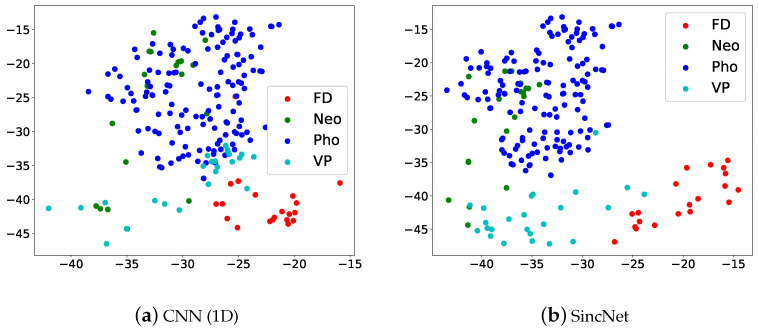
Scatter plots of (**a**) CNN(1D) and (**b**) SincNet show the t-SNE performance for classifying FD, Neo, Pho, and VP using FEMH dataset.

Figure 16 illustrates the t-SNE Clustering, Red dots are FD, green dots are Neo, blue dots are Pho and light blue dots are VP. We can see that the four kinds of voice disorders can be distinguished. But there is a partial overlap, which may also be verified from Figure 15. Results show that the SincNet outperforms CNN.

## 4. Discussion

**Figure 17:**
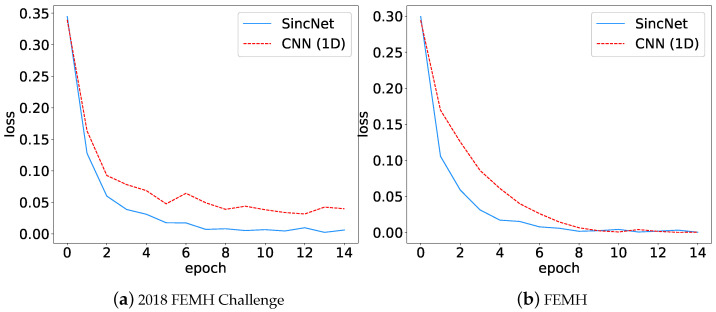
During training SincNet and CNN(1D) models in (**a**) 2018 FEMH Challenge and (**b**) FEMH datasets, lose curves were depicted in this figure to demonstrate the training efficiency.

The training efficiency for providing CNN(1D) and SincNet models are evaluated in terms of loss curves, where the results are illustrated in Figure 17. We evaluated (a) the 2018 FEMH Challenge and (b) the FEMH training sets. From both figures, the loss-decreasing rate of SincNet is higher than that of CNN(1D). The fast convergence rate shows that applying Sinc Filters in the first layer can increase the learning efficiency for the downstream classification and detection applications.

**Figure 18:**
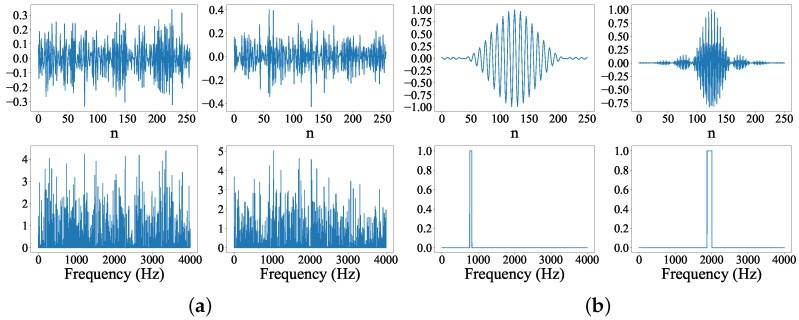
Two channels selected from the input layer of the optimized CNN(1D) and SincNet models were used to demonstrate the filter properties. The upper row shows filters in the time domain, while the bottom row depicts the magnitude components of filters in the frequency domain. (**a**) CNN Flters${}_{35,72}$; (**b**) Sinc Flters${}_{35,72}$.

All channels in the input layer were drawn from each optimized CNN(1D) (denoted as “CNN Filters”) and SincNet (denoted as “Sinc Filters”). We picked the 35-th and 72-th channels from “CNN Filters” (denoted as “CNN Filters${}_{35,72}$”) and the 35-th and 72-th filters from “Sinc Filters” (denoted as “Sinc Filters${}_{35,72}$”). Then, we depicted “CNN Filters${}_{35,72}$” and “Sinc Filters${}_{35,72}$” in Figure 18a,b, respectively. For each sub-figure in Figure 18a,b, the upper row represents temporal sequences of filters while the bottom one illustrates the corresponded magnitude trajectory in the frequency domain. From Figure 18a, “CNN Filters” provides a full-band filter in each channel for CNN(1D) to process the input signal. Conversely, the “Sinc Filters” in Figure 18b used a series of optimized band-pass filters for filtering the input voice sequences in a SincNet system. The above observations imply that the “Sinc Filters” can be used to extract more frequency-aware acoustic features for the following CNN/DNN model in Figure 2.

**Figure 19:**
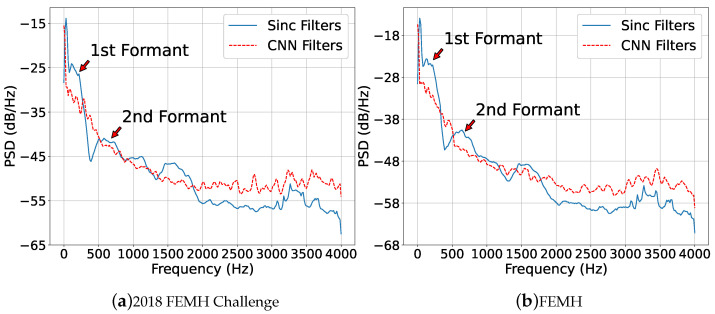
The power spectral density of “CNN Filters” and “Sinc Filters” processed pathological voices. Two utterances individually selected from (**a**) 2018 FEMH Challenge and (**b**) FEMH datasets were involved in this test.

To further demonstrate the “CNN Filters” and “Sinc Filters” processed pathological voices, we performed power spectral density (PSD) and depicted them in Figure 19. The PSD was calculated in the following procedure: (1) An /a/-vowel sound selected from a dataset was passed across “CNN Filters” or “Sinc Filters” to generate two different 80-dimensional feature sequences. (2) For an acoustic feature, the 256-point PSD operation was carried out for each dimensional signal and provided a PSD matrix with its size of 80×256. (3) Thereafter, we averaged all 80 trajectories to provide one PSD outcome. Notably, the PSDs in Figure 19a were depicted by processing an /a/-vowel utterance that was selected in the 2018 FEMH Challenge. Similarly, those PSDs in Figure 19b were made by processing the voice signal that was chosen from the FEMH database. Both figures show that the “Sinc Filters” processed speech signal can preserve more acoustic structures especially for the first formant, which is the major character of the /a/-vowel. for this study. Therefore, the SincNet system can introduce more accurate classification and detection performance for pathological speech than CNN(1D) provides.

## 5. Conclusions

This paper uses a series of learnable sinc functions to develop an explainable SincNet system for learning pathological voices. Unlike traditional CNN, the applied sinc filters, a front-end signal processor in SincNet, can construct the meaningful layer and are directly used to extract the acoustic features. We conducted our tests on three different Far Eastern Memorial Hospital voice datasets. The evaluation results demonstrate that the proposed SincNet system can effectively provide superior recognized accuracy and sensitivities in predicting input pathological waveforms, especially during small dataset conditions. Moreover, the proposed approach also improves convergence speed over a standard CNN and is more computationally efficient due to the exploitation of filter symmetry. Finally, we intended to give possible explanations between the system output and the first-layer extracted speech features based on our evaluated results.

## Figures and Tables

**Table 1:** For FEMH, the size of the pathological voices.

	FD	Neo	Pho	VP	Normal
/a/ sound	100	101	718	124	100

**Table 2:** For the 2018 FEMH-Challenge, the size of the pathological voices.

	Neo	Pho	VP	Normal
/a/ sound	50	50	50	50

**Table 3:** For the 2019 FEMH-Challenge, the size of the pathological voices.

	FD	Neo	Pho	VP
/a/ sound	100	100	100	100

**Table 4:** Detection performance for CNN(1D), CNN(2D), and SincNet on the 2018 FEMH Challenge dataset.

Model\Data	2018 FEMH Challenge			
	Sensitivity	Specificity	Accuracy	UAR
CNN(1D)	72.00% ± 7.16	65.00% ± 4.90	70.83 ± 3.84	68.50% ± 2.92
CNN(2D)	72.88% ± 5.66	62.35% ± 3.50	67.21 ± 2.52	67.61% ± 2.62
SincNet	**80.00% ± 4.97**	**65.00% ± 2.45**	**77.50 ± 2.68**	**72.50% ± 1.87**

**Table 5:** Detection performance for CNN(1D), CNN(2D), and SincNet on the FEMH dataset.

Model\Data	FEMH			
	Sensitivity	Specificity	Accuracy	UAR
CNN(1D)	77.88% ± 3.16	60.00% ± 3.90	76.32 ± 3.58	68.94% ± 2.78
CNN(2D)	74.68% ± 4.66	58.35% ± 3.55	73.45 ± 2.37	66.52% ± 2.59
SincNet	**84.62% ± 2.97**	**70.00% ± 1.64**	**83.33 ± 2.42**	**77.31% ± 1.75**

**Table 6:** Classification performance of Neo, Pho, and VP for CNN(1D), CNN(2D), and SincNet on the 2018 FEMH Challenge dataset.

Model\Data	2018 FEMH Challenge				
	Neo	Pho	VP	Accuracy	UAR
CNN(1D)	66.67% ± 2.30	67.24% ± 3.56	75.00% ± 2.98	69.00 ± 3.78	69.64% ± 2.65
CNN(2D)	60.00% ± 1.45	63.00% ± 2.66	65.00% ± 2.33	63.83 ± 2.72	62.67% ± 3.63
SincNet	**72.22% ± 1.55**	**70.69% ± 2.69**	**79.17% ± 2.17**	**73.00 ± 2.12**	**74.03% ± 2.57**

**Table 7:** Classification performance of Neo, Pho, and VP for CNN(1D), CNN(2D), and SincNet on the 2019 FEMH Challenge dataset.

Model\Data	2019 FEMH Challenge (Remove FD)				
	Neo	Pho	VP	Accuracy	UAR
CNN(1D)	60.00% ± 1.53	65.00% ± 2.65	65.00% ± 1.28	63.33 ± 3.42	63.33% ± 3.66
CNN(2D)	59.00% ± 1.69	66.45% ± 3.27	64.15% ± 2.96	63.13 ± 4.44	63.20% ± 2.19
SincNet	**75.00% ± 1.55**	**70.00% ± 2.37**	**65.00% ± 1.55**	**70.00 ± 1.63**	**70.00% ± 1.12**

**Table 8:** Classification performance of Neo, Pho, and VP for CNN(1D), CNN(2D), and SincNet on the FEMH dataset.

Model\Data	FEMH (Remove FD)				
	Neo	Pho	VP	Accuracy	UAR
CNN(1D)	80.00% ± 2.90	78.32% ± 3.16	75.00% ± 2.68	78.07 ± 2.75	77.77% ± 2.56
CNN(2D)	78.00% ± 2.45	90.00% ± 3.66	50.00% ± 2.55	76.63 ± 1.58	72.67% ± 2.62
SincNet	**75.00% ± 1.35**	**81.82% ± 2.68**	**83.33% ± 2.43**	**81.28 ± 1.48**	**80.05% ± 1.29**

**Table 9:** Classification performance of FD, Neo, Pho, and VP for CNN(1D), CNN(2D), and SincNet on the 2019 FEMH Challenge dataset.

Model\Data	2019 FEMH Challenge					
	FD	Neo	Pho	VP	Accuracy	UAR
CNN(1D)	75.00% ± 2.30	55.00% ± 3.56	70.00% ± 2.98	50.00% ± 2.65	62.50 ± 2.00	62.50% ± 3.43
CNN(2D)	75.00% ± 1.45	50.00% ± 2.66	65.00% ± 2.33	50.00% ± 3.63	59.45 ± 2.12	60.00% ± 2.33
SincNet	**75.00% ± 1.55**	**65.00% ± 2.69**	**75.00% ± 2.17**	**60.00% ± 2.57**	**68.75 ± 1.32**	**68.75% ± 1.50**

**Table 10:** Classification performance of FD, Neo, Pho, and VP for CNN(1D), CNN(2D), and SincNet on the FEMH dataset.

Model\Data	FEMH					
	FD	Neo	Pho	VP	Accuracy	UAR
CNN(1D)	60.00% ± 2.00	55.00% ± 2.48	68.53% ± 3.70	54.17% ± 2.66	64.73 ± 3.29	59.43% ± 2.00
CNN(2D)	55.00% ± 2.48	80.00% ± 2.94	40.00% ± 3.83	50.00% ± 2.32	60.24 ± 2.45	56.25% ± 2.03
SincNet	**50.00% ± 2.00**	**65.00% ± 1.48**	**75.52% ± 2.27**	**66.67% ± 2.48**	**71.01 ± 1.17**	**64.30% ± 1.32**

## Data Availability

Due to its proprietary nature <or ethical concerns>, supporting data cannot be made openly available.
